# An Ultra-Thin and Wideband Low-Frequency Absorber Based on Periodic Resistance Film

**DOI:** 10.3390/ma19081577

**Published:** 2026-04-14

**Authors:** Tianjiao Bao, Pengrui Liu, Tong Zhang, Haosen Wang, Yafa Zhang

**Affiliations:** AECC Beijing Institute of Aeronautical Materials, Huanshan Village, Wenquan Town, Haidian District, Beijing 100095, China

**Keywords:** periodic resistive layer, ultra-thin absorber, low-frequency broadband absorption, impedance matching, electromagnetic loss

## Abstract

Low-frequency broadband electromagnetic wave absorption is a critical challenge for radar stealth materials, as traditional absorbent-based coatings often suffer from poor low-frequency performance or severe high-frequency degradation when optimized for low frequencies. This study proposes a novel ultra-thin broadband low-frequency absorber fabricated by depositing a periodic resistive layer onto a conventional absorbent-based wave-absorbing layer, which forms a tailored low-frequency conductive metasurface structure. The integrated coating achieves an ultra-thin total thickness of merely 0.4 mm while exhibiting excellent broadband absorption performance across multiple radar bands: it delivers an average reflection loss of −0.6 dB in the L-band (1–2 GHz), −2 dB in the S-band (2–4 GHz), −3.6 dB in the C-band (4–8 GHz), and maintains a stable average reflection loss of −2.8 dB in the X to Ku bands. Compared with single-layer absorbing materials of the same thickness, this material exhibits significantly improved absorbing performance in the S-band and C-band, and achieves a breakthrough from zero to effective absorption in the L-band. Meanwhile, it can be integrated with structural design to reduce radar cross section (RCS), showing excellent engineering application value. The key mechanism underlying the performance enhancement lies in the periodic resistive layer, which optimizes the broadband impedance matching of the entire coating system, effectively elevates the surface current density, and augments resistive loss and eddy current loss within the structure. This design strategy enables an effectively boost in S-band wave-absorbing performance with minimal compromise to the high-frequency absorption characteristics, thus meeting the stringent requirements for broadband radar wave absorption in practical engineering applications.

## 1. Introduction

With the continuous expansion of radar wave frequency bands, low-frequency electromagnetic waves, particularly decimeter and meter waves, have assumed an increasingly pivotal role in military applications [1,2,3]. Electromagnetic waves with shorter wavelengths offer superior resolution yet suffer from poor penetration, which consequently restricts their detection range. In contrast, longer-wavelength decimeter and meter waves possess exceptional penetration capability, enabling the identification of long-distance targets concealed or camouflaged by various obstructions, and thus facilitating the detection of short-, medium-, and long-range ballistic missiles. In modern military operations, low-frequency electromagnetic wave absorbing materials (LFAMs) for countering meter-wave radar detection have become indispensable for the long-distance camouflage of weapon systems [4,5]. Therefore, research on LFAMs holds profound military significance. Stealth aircraft are coated with wave-absorbing materials on their fuselages, which convert incident radar electromagnetic waves into thermal energy when the waves impinge on the surface, and this process drastically reduces the reflection loss of electromagnetic waves [6]. Hence, enhancing wave-absorbing performance across the entire radar frequency spectrum—especially in the long-wavelength band—has emerged as the prevailing development trend for wave-absorbing materials.

Ultra-thin wave-absorbing coatings are extensively utilized as radar absorbing materials (RAMs) owing to their notable advantages, including no modification to the structural design of the substrate material, low manufacturing cost, and minimal weight increment. To date, researchers have made substantial progress in the utilization of magnetic materials (MMs) for low-frequency electromagnetic wave absorption. MM-based absorbers exhibit excellent electromagnetic wave absorption performance in the X-band [7,8]; for example, wave-absorbing coatings fabricated with magnetic absorbents such as carbonyl iron can maintain a compact thickness while achieving high absorption efficiency in this band. However, constrained by the intrinsic properties of absorbents and coating thickness, such absorbers exhibit nearly negligible absorption performance at low frequencies. Electromagnetic waves in the L-band (1–2 GHz) and S-band (2–4 GHz) have long wavelengths, rendering them resistant to attenuation by traditional electromagnetic wave absorbers [9,10]. To enhance the low-frequency performance of homogeneous radar wave-absorbing coatings, the mainstream design strategy involves employing absorbents with high permittivity and high magnetic permeability (e.g., flake-shaped low-frequency absorbents such as FeAlSi alloys) to tailor low-frequency absorption characteristics. Nevertheless, the spectral performance of powder-based absorbents is inherently limited by their material composition, which makes it challenging to simultaneously optimize low- and high-frequency absorption performance. Specifically, the improvement of low-frequency absorption is invariably accompanied by a significant degradation in high-frequency performance, resulting in poor broadband absorption performance. Traditional dielectric loss-dominated absorbers also suffer from the drawbacks of large thickness and narrow operating bandwidth in the low-frequency band due to their intrinsic material limitations. Accordingly, the development of high-performance microwave absorbers operating at the S-band and lower frequencies remains a major technical challenge. In recent years, the integration of MMs with metamaterials to further extend the low-frequency operating bandwidth of microwave absorbers has attracted extensive research attention [11,12]. Shou et al. [13] proposed an S-band ultra-thin metamaterial absorber based on magnetic materials, achieving < −10 dB reflection coefficient (≥90% absorptivity) over 1.73–4.04 GHz (80.1% fractional bandwidth) with a total thickness of 3.4 mm, consisting of a centrosymmetric open square ring and a central circular patch. Mehta [14] developed a broadband metamaterial absorber (0.32 cm thick) with two absorption bands (3.7–5.6 GHz and 5.92–9.26 GHz) and a peak absorptivity of 99.98% at 8.86 GHz, composed of diagonal C-shaped patterns, an epoxy substrate, and a metallic back plate. Elakkiya et al. [15] reported a five-band polarization-insensitive absorber with an ultra-thin 0.014λ FR4 substrate, exhibiting absorptivity of 98–99.9% at 5.4 GHz (C-band), 8.7 GHz (X-band), and 14.53–15.68 GHz (Ku-band). Ning et al. [16] designed a tunable low-frequency absorber (5 mm thick) using varactors and magnetic nanomaterials, achieving a continuously tunable 0.41–1.02 GHz band (85.3% fractional bandwidth) with ≥10 dB reflection reduction. Patel [17] presented a 1.6 mm-thin FR4-based absorber with dual absorption peaks at 3 GHz (S-band) and 6.9 GHz (C-band), average absorptivity > 90%, and angle stability. Zhang et al. [18] proposed a magnetic metamaterial absorber with a needlepoint pattern, expanding bandwidth by >65% via LC resonances and dominated by magnetic loss. Lin [19] developed a triple-band low-frequency ultra-compact absorber (10.92 mm unit cell width) with 96.5–99.6% absorptivity at 2.151–2.378 GHz, constructed from 3D periodic structures on lossy FR4-epoxy. While these studies demonstrate effective low-frequency absorption via metamaterial design, most reported structures feature relatively large thicknesses, limiting their direct integration into existing aircraft structures.

It should be emphasized that the actual electromagnetic absorption performance and long-term durability of stealth coatings are often significantly affected by environmental factors, including temperature, humidity, and air pollution.

Moreover, the electrical and physical properties of the underlying substrate also exert a non-negligible influence on the impedance matching and overall absorption behavior of such coatings.

In this study, a novel wave-absorbing pattern was designed by introducing a periodic resistive layer on an absorbent-based wave-absorbing layer, and this design constructs a low-frequency conductive structure. This structural design effectively improves the low-frequency wave-absorbing performance to a considerable extent without exerting a significant adverse effect on the high-frequency band. At an ultra-thin thickness of only 0.4 mm, the coating achieves an average reflection loss of −0.6 dB in the L-band, −2 dB in the S-band, −3.6 dB in the C-band, and maintains an average reflection loss of −2.8 dB in the X- to Ku-bands. The material exhibits improved performance in the S-band and C-band, outperforming single-layer absorbing materials with the same thickness. The designed periodic resistive layer can optimize the broadband impedance matching of the coating, thereby enhancing the surface current density, augmenting resistive loss and eddy current loss, which allows the enhancement of low-frequency absorption performance with minimal degradation in high-frequency performance, thus satisfying the requirements for broadband absorption characteristics.

## 2. Materials and Methods

**Figure 1:**
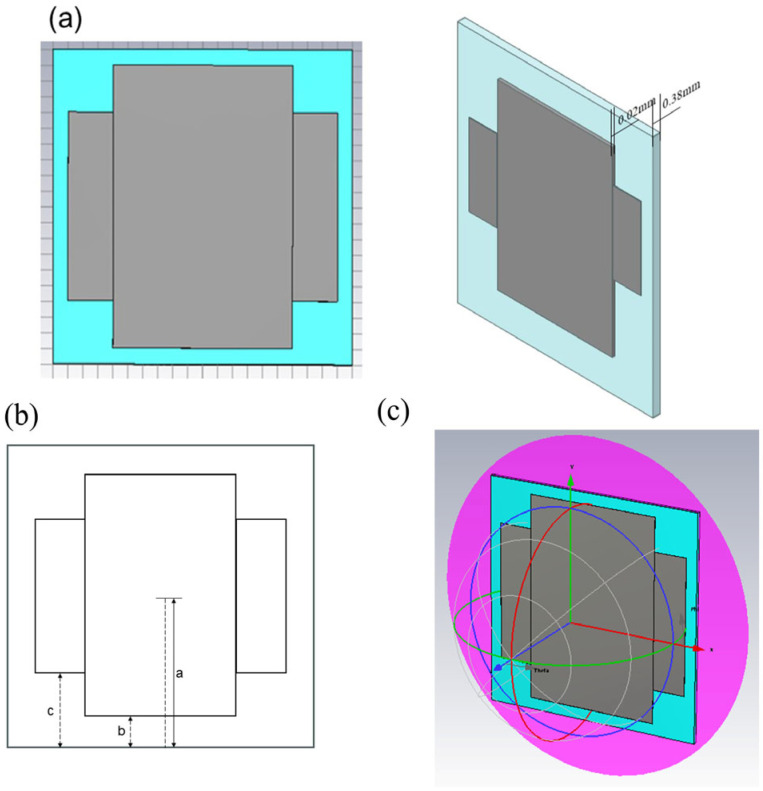
The meta-material absorber (**a**) the design structure (**b**) the size of the structure (**c**) the source of simulation.

**Figure 2:**
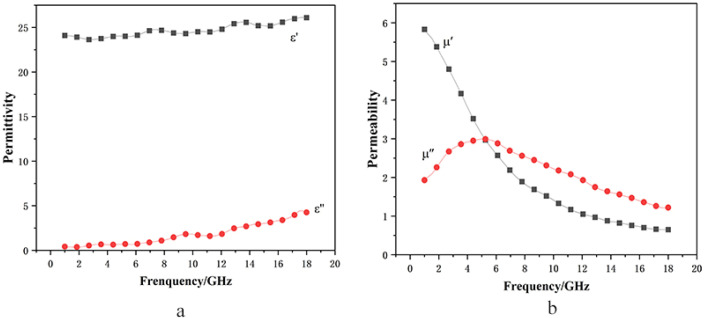
Electromagnetic parameters of carbonyl iron absorbents ((**a**) real and imaginary parts of dielectric constant; (**b**) real and imaginary parts of magnetic permeability).

The design of the absorbing material consists of a two-layer structure (Figure 1). The top layer is an ultra-thin periodic patterned layer, whose unit cell structure is shown in Figure 1a. It is fabricated using a resistive film with a sheet resistance of 100 Ω/sq, and the pattern is designed as a symmetric cross shape. The size of the structure is shown in Figure 1b. The length of each cross arm is 2(a − b), and the width of each cross arm is 2(a − c), with the design parameters of a = 10 mm, b = 1 mm, and c = 4 mm. The thickness of the resistive film is 0.02 mm. The bottom layer is a continuous resin-based wave-absorbing coating, which uses commercial carbonyl iron as the absorbent with a material thickness h_1_ = 0.38 mm. The complex permittivity (ε_r_) and complex permeability (μ_r_) of carbonyl iron were measured by a vector network analyzer, and the results are presented in Figure 2. The backplate is modeled as a perfect electric conductor (PEC).

Numerical simulations are performed using the commercial electromagnetic simulation software CST Microwave Studio 2022, Dassault Systèmes, Vélizy, France, with the frequency-domain solver. Unit cell boundary conditions were applied in the X and Y directions to represent an infinite periodic array, and open (add space) boundaries were set along the Z-axis for free-space radiation. Floquet ports were used to excite normal-incidence plane waves propagating along the Z-axis direction, as depicted in Figure 1c. A sufficiently refined mesh was adopted to ensure convergence and numerical stability of the simulated results. Under such configuration, S11 (in dB) is used to characterize the reflection loss (RL) in the research of microwave absorbing materials, which is obtained by calculating 20 times the logarithm of the absolute value of the reflection coefficient S11, which is derived from the ratio of the reflected wave amplitude to the incident wave amplitude at the input port.

To verify the design scheme, a metamaterial microwave absorption test panel was fabricated. In accordance with the design protocol, the first layer was a homogeneous microwave-absorbing coating with carbonyl iron absorbents as the absorbent, featuring a thickness of 0.38 mm, which is denoted as the homogeneous microwave absorbing material. The carbonyl iron absorbent was the same as the simulation. Furthermore, a 0.02 mm-thick carbon-based patterned layer was deposited on the homogeneous microwave absorbing coating, where the sheet resistance of the patterned area was controlled at 100 Ω/sq, and the pattern dimensions were consistent with those in the simulation design. An arched frame apparatus was adopted to measure the S11 parameter of the absorber to obtain reflection loss.

## 3. Results

### 3.1. Simulation

**Figure 3:**
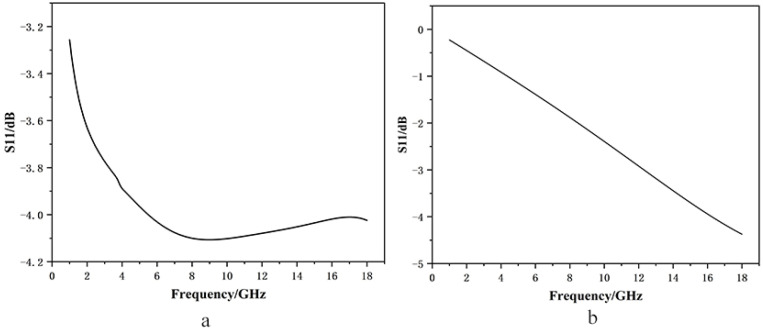
Simulated reflection loss of microwave absorbing materials: (**a**) with resistance film and (**b**) without resistance film.

The simulation results are presented in Figure 3a. The results indicate that the reflection loss of the structure remains below −4 dB over a broad frequency range of 5–18 GHz, with the strongest absorption occurring at 8 GHz. For comparison, the reflection loss of the structure with only the single homogeneous absorber coating (i.e., without the resistive film) was also simulated. The result of absorption was shown in Figure 3b. As can be observed from Figure 3a, the introduction of the resistive film significantly reduces the reflection loss in the low-frequency region, thereby effectively enhancing the low-frequency absorption performance.

For the single-layer absorber, the resonant frequency of the material is above 18 GHz due to the ultra-thin coating thickness. When the resistive film layer is incorporated, multi-resonance coupling is realized, which satisfies the conditions for broadband absorption. The employment of the resistive film contributes to improved low-frequency absorption. Meanwhile, owing to the relatively large gaps between the patterned elements, high-frequency electromagnetic waves can penetrate these gaps, which minimizes the adverse effect on the high-frequency response and preserves the excellent high-frequency absorption performance of the base layer.

**Figure 4:**
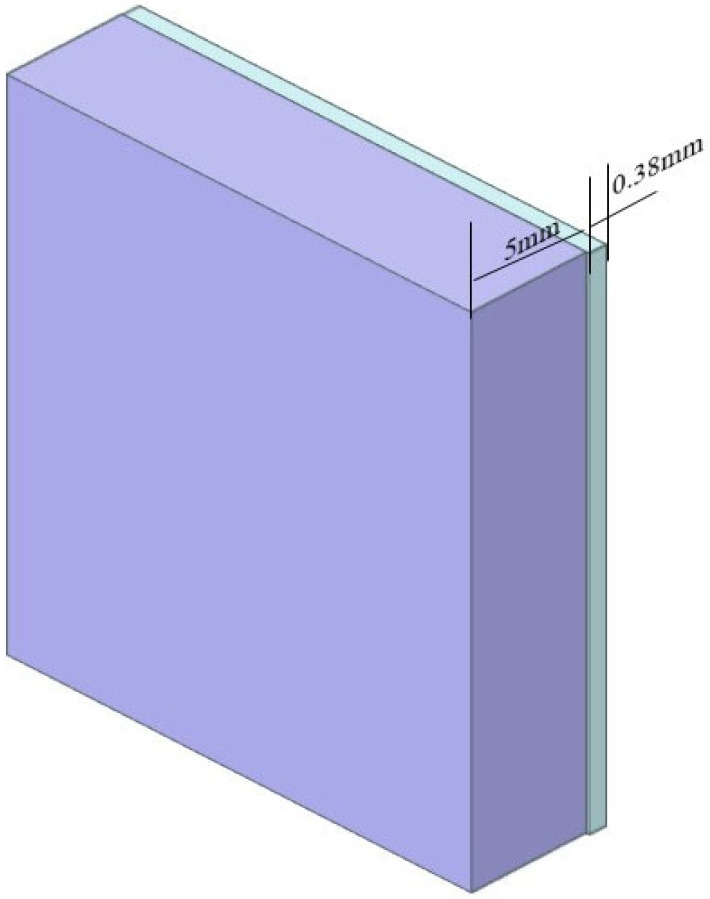
The 3D model of absorber structure.

**Figure 5:**
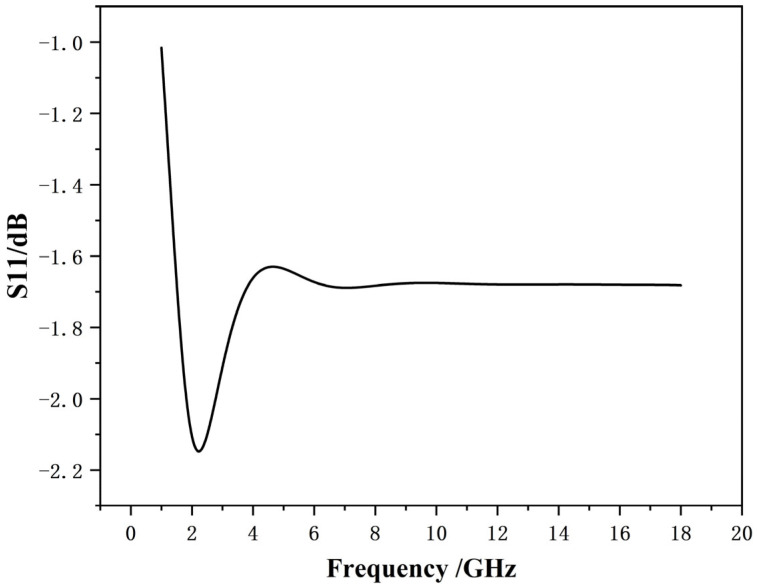
The simulation results of reflection loss of absorbing material with low frequency absorber.

Conventionally, low-frequency absorbers are adopted to optimize the low-frequency absorption performance of absorbing coatings. To compare with the proposed resistive film strategy, we also simulated a homogeneous coating design using low-frequency absorbers. The wave absorption performance of the material was simulated using electromagnetic simulation software, and the material thickness was optimized to achieve satisfactory low-frequency absorption, The proposed structure is illustrated in Figure 4, and the reflection loss characteristics are presented in Figure 5. The reflection loss in the L-band reaches only −2 dB when the material thickness exceeds 5 mm, whereas the absorption performance in the X–Ku bands degrade drastically, with the reflection loss increasing to approximately −1.7 dB. Further increasing the thickness of the low-frequency layer will lead to an even greater deterioration in high-frequency performance. These results demonstrate that thin films fabricated from homogeneous wave-absorbing materials require a much larger thickness to realize effective low-frequency absorption, accompanied by a significant sacrifice of high-frequency absorption, thus failing to achieve broadband absorption.

### 3.2. Impedance Analysis

**Figure 6:**
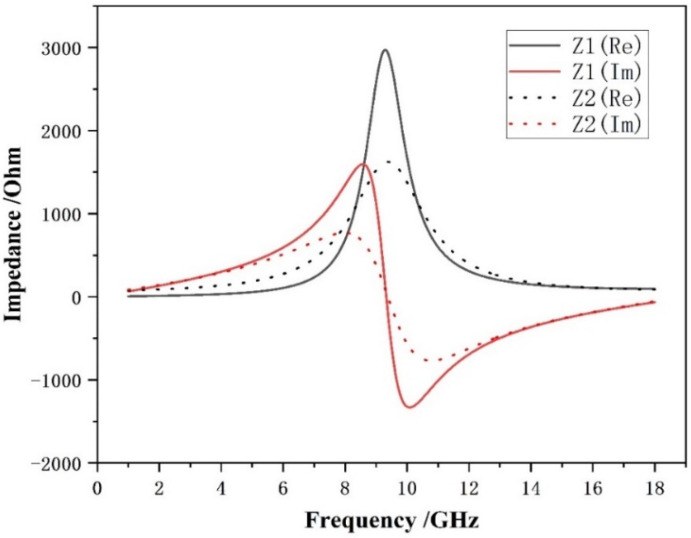
Real and imaginary parts of impedance for microwave absorbing materials: Z1 (without resistance film) and Z2 (with resistance film).

To gain insight into its working mechanism, we analyze the impedance matching properties of the entire absorber. Owing to the metallic backplate, the S21 parameter is zero, which simplifies the impedance expression (Z) to Equation (1) [20]; the corresponding relative impedance profile is accordingly presented in Figure 6.

(1) $$Z=\frac{1+S_{11}}{1-S_{11}}$$

Impedance matching analysis shows that, as illustrated in the Figure 6, the solid lines represent the real and imaginary parts of the impedance of the second layer without the resistive film, both of which differ significantly from the free-space impedance (377 Ω). After introducing the resistive film, the real and imaginary parts of the coating impedance become much closer to the free-space impedance over a broad frequency range, demonstrating that the addition of the resistive film improves impedance matching.

The core mechanism of high low-frequency absorption enabled by the resistive film lies in the resistive loss of the material itself, combined with structural design to realize broadband impedance matching. In particular, this strategy overcomes the dimensional challenges caused by the long wavelengths of low-frequency electromagnetic waves. For low-frequency waves with long wavelengths, resonant-type absorbing structures require unit cells comparable in size to the operating wavelength, resulting in bulky and impractical configurations. Carbon-based coating resistive films possess an appropriate electrical resistivity. When electromagnetic waves are incident, the alternating electric field drives the motion of free electrons or charge carriers, which collide with the crystal lattice and directly convert electromagnetic energy into Joule heat. To achieve high absorption, electromagnetic reflection at the material surface must be minimized to allow more energy to penetrate into the interior. By optimizing the sheet resistance of a single resistive film, its equivalent wave impedance can be tuned to approach the free-space impedance (377 Ω), thereby achieving impedance matching and minimal reflection at specific frequencies.

### 3.3. Surface Current Distribution

We further analyzed its surface current at each peak of the absorption band. The analysis of the current and electric field distributions provides an intuitive visualization of the electromagnetic wave absorption effect induced by the resistive film. The outcomes are presented below.

The resistive film design can effectively optimize the surface impedance matching, thereby enabling more electromagnetic energy to be coupled into the material and inducing a stronger surface current within the absorber. Due to the electrical conductivity of the resistive film, the incident electromagnetic waves first excite a conduction current on the surface of the absorber. At the resonant frequency of 1 GHz, the absorber is well-matched to the incident wavelength, leading to the in-phase superposition of the current along specific structural paths and a sharp increase in current intensity.

**Figure 7:**
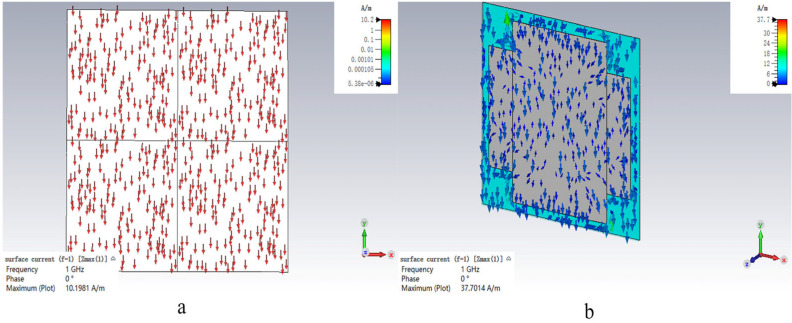
Surface current distribution of microwave absorbing materials: (**a**) without resistance film and (**b**) with resistance film.

As observed from the current distribution maps in Figure 7, at f = 1 GHz, the maximum surface current density is only 10 A/m in the absence of the resistive film. In contrast, after introducing the resistive film, the surface current density is significantly enhanced, reaching up to 37.7 A/m. The absorber material itself possesses a finite conductivity (non-ideal conductor). According to Joule’s law, P = I^2^R, the Joule heating power is proportional to the square of the current intensity (I). Therefore, the regions with high current density shown in the figures (especially the red regions close to 37.7 A/m) represent the dominant “hot regions” where electromagnetic energy is converted into thermal energy.

When the current flows through the resistive component of the material, direct Joule heating occurs, dissipating the incident electromagnetic energy. At resonance, charges accumulate in regions with high electric field intensity; these concentrated electric fields drive the motion of charges and form electric currents. The concentrated current further generates thermal dissipation through the material resistance.

In addition, the alternating surface current induces a closed time-varying magnetic field. This time-varying magnetic field can further generate eddy currents inside the absorber or within adjacent conductive layers. The loop-like or vortex-like distribution of the current observed in the figures is a direct manifestation of this effect. As these eddy currents propagate through the resistive material, additional Joule heat is generated, accompanied by magnetic hysteresis loss and eddy current loss, providing another pathway for converting electromagnetic energy into thermal energy. Accordingly, a higher current density gives rise to stronger dielectric loss and eddy current loss. The maximum current density in the current field is a key indicator for evaluating the resonance intensity and the potential absorption capability. A higher value indicates a greater energy density captured and ready to be dissipated.

### 3.4. Parameters Analysis

In the design of meta surfaces, the electromagnetic parameters need to be optimized. We further explored the structural parameters of the designed pattern and investigated the influences of different parameters on the microwave absorption performance.

**Figure 8:**
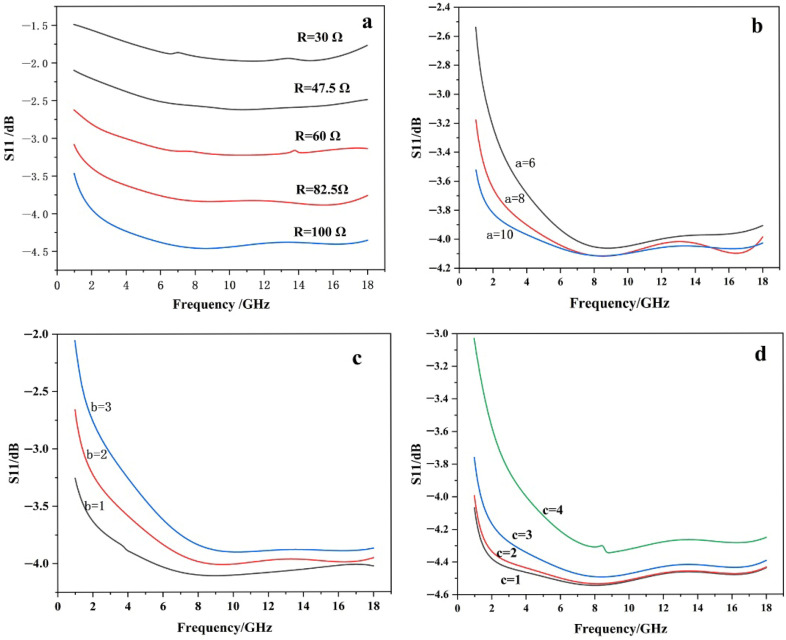
Simulated reflection loss (RL) for resistance film patterns with different structural parameters: (**a**) different sheet resistances R; (**b**) different geometric parameter a; (**c**) different geometric parameter b; (**d**) different geometric parameter c.

In the cross-shaped pattern, the arm length and arm width of the cross are the key structural parameters, which are mainly determined by the variables a, b, and c in the diagram. As the value of a increases and the value of c decrease continuously, the feature size of the pattern in the resistive film increases as shown in Figure 8, which improves the low-frequency absorption performance of the material, resulting in better microwave absorption.

As the sheet resistance increases, the overall reflection loss decreases as shown in Figure 8a. At a low sheet resistance, the enhanced reflection effect induced by the resistive film degrades the impedance matching, thereby reducing the absorption performance.

### 3.5. Experiment

**Figure 9:**
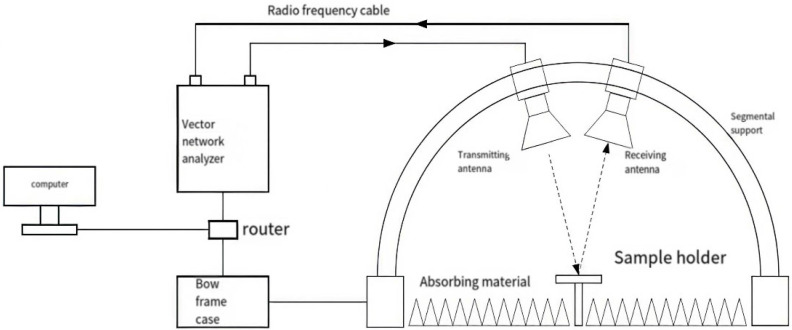
Schematic of reflection loss measurement via arched frame method.

**Figure 10:**
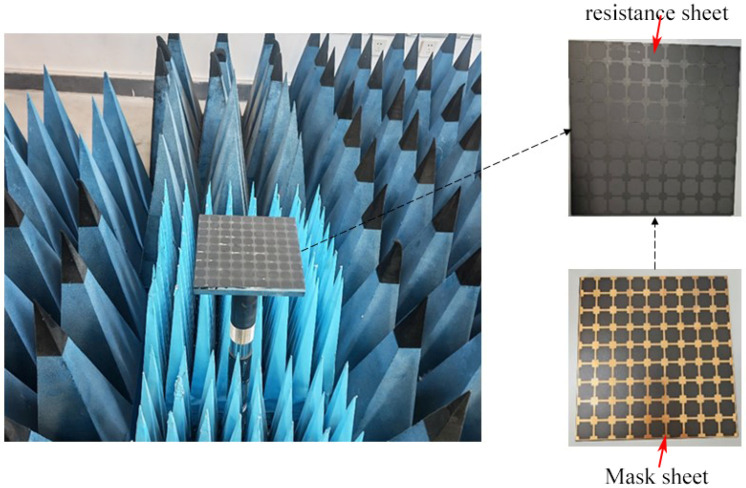
Actual measurement image of reflection loss test by arched frame method.

To verify the design scheme, a metamaterial microwave absorption test panel was fabricated to test its actual microwave absorption performance, as shown in Figure 9 and Figure 10.

**Figure 11:**
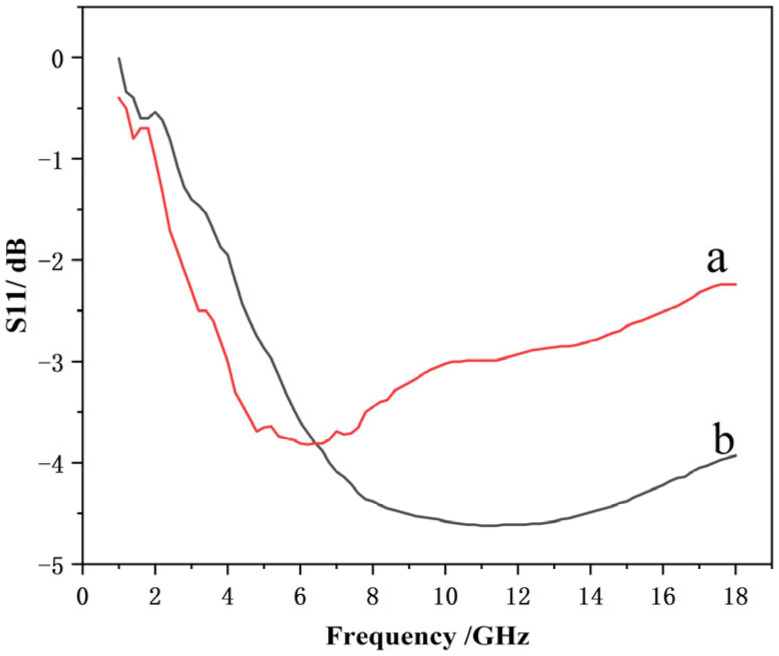
Measured reflection loss of microwave absorbing materials: (**a**) with resistance film and (**b**) without resistance film.

The experimental results (Figure 11) exhibit the same trend as the simulation results, which indicates that this design strategy is a feasible approach to enhance the low-frequency microwave absorption performance. Specifically, with an ultra-thin total thickness of 0.4 mm, the microwave absorbing material achieves an average reflection loss of less than −0.6 dB in the L-band, less than −2 dB in the S-band, less than −3.6 dB in the C-band, and maintains an average reflection loss of less than −2.8 dB in the X–Ku bands. Such low-frequency performance is hardly achievable with a conventional 0.4 mm-thick microwave absorbing coating. For the conventional carbonyl iron powder (CIP) microwave absorbing material, at a thickness of 0.4 mm, the absorption level in the X–Ku bands can reach below −3 dB, while almost no absorption performance is observed in the L- and S-bands as a comparison in Figure 11. Based on previous experience, after low-frequency powder fillers are added, when the low-frequency performance is improved to approximately −0.5 dB, the reflection loss in the X-band will increase to above −2 dB.

By comparison with the relevant literature in Table 1, other studies have also improved low-frequency absorption performance through metamaterial surface design. Nevertheless, the thickness of the reported structures is generally above 1 mm, and no comparable coating structure as thin as 0.4 mm has been reported. This indicates that the proposed structure in this work presents certain advancement in achieving both low-frequency absorption and ultra-thin thickness. Such an ultra-thin coating of only 0.4 mm possesses significant engineering significance for applications in special restricted spaces and specific installation positions.

However, the actual wave-absorbing performance is inconsistent with the design scheme, indicating potential for further optimization. The simulated average reflection loss is −3.5 dB in the L-band, below −3.7 dB in the S-band, approximately −4 dB in the C-band, and remains below −4 dB on average in the X–Ku bands. By contrast, the measured average reflection loss is below −0.6 dB in the L-band, below −2 dB in the S-band, below −3.6 dB in the C-band, and below −2.8 dB in the X–Ku bands, which is overall weaker than the simulated performance. Notably, the deviation between simulation and measurement is most significant at the low-frequency L-band, relatively moderate in the S- and C-bands, and becomes pronounced again at high-frequency X–Ku bands.

At low frequencies, the absorption performance strongly relies on precise impedance matching, which is extremely sensitive to the effective electromagnetic parameters of the carbonyl iron coating and the sheet resistance uniformity of the resistive film. Slight fluctuations in the content and dispersion of magnetic particles will evidently degrade the low-frequency loss and matching condition. In addition, parasitic parameters introduced by the test fixture and sample boundary also exhibit a more evident influence at lower frequencies.

At high frequencies (X–Ku bands), the short wavelength makes the structure highly sensitive to fabrication tolerances, surface roughness, and interlayer contact gaps. Dimensional errors of the metamaterial pattern and local inhomogeneity of the coating lead to impedance mismatch, resonant peak deviation, and weakened absorption.

By comparison, the S- and C-bands present better consistency, as the moderate wavelength reduces the sensitivity to both parasitic effects and machining errors.

Other non-negligible factors include the deviation between the actual sheet resistance of the resistive film and the ideal design value, uneven distribution and agglomeration of carbonyl iron particles, as well as the finite-size effect of the actual sample compared with the infinite periodic model in simulation. All these factors collectively lead to the observed differences in reflection loss magnitude and absorption peak characteristics across different frequency bands.

The experimental results confirm that employing a resistive film with appropriately designed parameters as the surface matching layer of the microwave absorbing material can effectively improve the low-frequency performance without significantly increasing the material thickness, which is conducive to practical engineering applications. In addition, commercial software can provide effective guidance for the design of microwave absorbing structures and possesses considerable guiding significance in engineering practice, thereby verifying the feasibility of the proposed design scheme.

## 4. Conclusions

In this paper, a thin metamaterial absorber based on a cross structure is designed and fabricated. Simulation results show that the absorber, with a total thickness of only 0.4 mm, achieves an absorption band ranging from 1 GHz to 18 GHz with a reflection loss below −3 dB. A comparative analysis was conducted between the proposed metamaterial coating and homogeneous low-frequency absorbers. To achieve comparable absorption performance, the required thickness of homogeneous wave-absorbing coating is impractically large. The impedance and surface current distribution are systematically analyzed. Results demonstrate that impedance tuning via the surface resistive layer optimizes the overall impedance matching of the material and increase the surface current density, thereby boosting absorption performance. Specifically, the low-frequency absorption performance of the material is continuously improved with an increase in the feature size of the surface resistive film and the sheet resistance of the resistive film. The design scheme of the metamaterial surface is further validated through experimental fabrication and testing. At an ultra-thin thickness of 0.4 mm, the measured average reflection loss of the coating is −0.6 dB in the L-band, −2 dB in the S-band, and −3.6 dB in the C-band; meanwhile, an average reflection loss of −2.8 dB across the X- to Ku-bands is maintained. This work achieves enhanced broadband absorption performance with an ultra-thin thickness of merely 0.4 mm, rendering it highly promising for practical engineering applications. Although effective absorption is realized in the 1–18 GHz band, the low-frequency range (particularly below 1 GHz) still holds substantial room for improvement. Combining the patterned resistive film design with other resonant structures (e.g., spiral resonators, split-ring resonators) can effectively overcome the inherent physical constraint of low-frequency absorption typically requiring a large material thickness. Importantly, based on the established simulation structure, a critical focus for future improvement lies in engineering implementation: accurate testing of the electromagnetic parameters of each dielectric layer, as well as strict control over thickness uniformity and material dispersion uniformity, is essential to better translate the simulated results into practical performance. These engineering control measures, together with other auxiliary research directions, lay a solid foundation for the practical application of ultra-thin metamaterial absorbers optimized for low-frequency performance.

## Figures and Tables

**Table 1:** Comparison of the performance of the proposed absorber with other reported absorbers.

Reference	Absorption Band	Thickness	RL	Design Structure
[21]	2.8–6.2 GHz	1 mm	−10 dB	Coating + metasurface
[22]	2.6–15.79 GHz	8 mm	−10 dB	Coating + metasurface
[23]	2–18 GHz	1.4 mm	−4 dB	Coating
proposed	2–4 GHz 4–8 GHz	0.4 mm	−2 dB −3.6 dB	Coating + metasurface

## Data Availability

The original contributions presented in this study are included in the article. Further inquiries can be directed to the corresponding author.
